# Copper Isotope Evidence of Oxidative Stress–Induced Hepatic Breakdown and the Transition to Hepatocellular Carcinoma

**DOI:** 10.1016/j.gastha.2022.02.024

**Published:** 2022-04-19

**Authors:** Philippe Telouk, Marie-Laure Plissonnier, Philippe Merle, Fabien Zoulim, Nadim Fares, Paule Guilloreau, Romain Parent, Justine Bacchetta, Marc Danan, Sergio Carandina, Francis Albarède

**Affiliations:** 1 Université de Lyon, ENSL, UCBL, CNRS, LGL-TPE, Lyon, France; 2 Cancer Research Center of Lyon (CRCL), UMR Inserm 1052 - CNRS 5286, Université Claude Bernard Lyon1, Lyon, France; 3 Department of Hepatology, Hospices Civils de Lyon, Croix-Rousse Hospital, Lyon, France; 4 Rangueil Hospital, CHU Toulouse, Toulouse, France; 5 Centre de Référence des Maladies Rares du Calcium et du Phosphore, Hôpital Femme Mère Enfant, INSERM 1033, Faculté de Médecine Lyon Est, Lyon, France; 6 Clinique Saint Michel, Société CCO, Toulon, France

**Keywords:** Copper Isotopes, Liver, Hepatocellular Carcinoma, Hypoxia

## Abstract

**Background and Aims:**

Hepatocellular carcinoma (HCC) is the third leading cause of cancer-related death worldwide, and finding a single reliable biomarker to follow liver degradation is a challenging task. To document the relationship between liver failure, hypoxia, and HCC, copper isotope variations (δ^65^Cu) were evaluated in the serum of HCC-negative and HCC-positive patients as a biomarker of hepatic failure.

**Methods:**

We analyzed Cu isotope variations in serum samples from 293 patients with potentially degraded liver functions presenting hepatitis B virus, hepatitis C virus, nonalcoholic steatohepatitis, and alcohol uptake (OH) etiologies and 105 controls. Ninety-five of the patients were diagnosed with HCC.

**Results:**

On average, the δ^65^Cu values of the serum of patients with F3-F4 fibrosis score or HCC-positive are low. The Cu isotope data are strikingly bimodal with well-defined δ^65^Cu modes which imperfectly reflect etiology. The population with normal values (ca −0.3‰) is progressively replaced by a population with atypical δ^65^Cu values (ca −0.8‰), which reflects the progressive degradation of hepatic functions.

**Conclusion:**

The clear bimodality does not correspond to a progressive shift of the δ^65^Cu values but to a replacement of one population by another. This bimodality sheds light on the persisting difficulties epitomized by α-fetoprotein in finding high-sensitivity and high-specificity HCC biomarkers. It is interpreted as a switch in the resistance of hepatic tissues to the oxidative stress that eventually leads to HCC oncogenesis.

## Introduction

Hepatocellular carcinoma (HCC), the sixth most common cancer and the third most common cause of cancer-related deaths, is often diagnosed at a late stage .^1^ The disease is driven by inflammation and is in most cases preceded by hepatitis virus infection and cirrhosis.^2^^,^^3^ Biomarkers with high sensitivity and specificity at the early stage of the disease are still missing.^4^, ^5^, ^6^, ^7^

Copper forms redox-active metal ions Cu(I/II) which are used as a cofactor of multiple enzymes involved in cellular respiration (mitochondrial cytochrome c oxidase), the control of reactive oxygen species (superoxide dismutase), and, in blood plasma, iron oxidation and angiogenesis (ceruloplasmin).^8^^,^^9^ Copper has 2 isotopes, ^63^Cu and ^65^Cu, with relative abundances particularly sensitive to the redox conditions prevailing in cells and body fluids as well as to bond energy and coordination number with various ligands, such as histidine and cysteine. Isotopic variations have been identified in all the compartments of biological activity.^10^, ^11^, ^12^, ^13^, ^14^, ^15^, ^16^ The Cu isotopic results are reported as conventional delta values, δ^65^Cu, which are the deviation in parts per 1000 of the ^65^Cu/^63^Cu ratio of the sample from that of the international reference material NIST SRM 976:

(C65uC63u)sample=(C65uC63u)SRM 976(1+δC65u1000)

The potential of transition-metal isotope compositions for the prognosis and diagnosis of diseases for which the liver is involved, directly or indirectly, such as hemochromatosis,^17^ Wilson disease,^18^ HCC,^12^, ^13^, ^14^^,^^19^ nonalcoholic fatty liver disease (NAFLD),^9^^,^^19^^,^^20^ breast and colon cancer,^15^^,^^21^ and myeloma,^22^^,^^23^ has recently emerged. A study by Bondanese et al.^24^ demonstrated that hypoxia caused measurable changes in the Cu isotopic composition of cultured HepG2 cell lines. The aim of the present work is to evaluate the usefulness of copper isotopic compositions in serum as a biomarker of the liver status in different types of common liver diseases and cancer in particular. A substantial number of cirrhosis cases evolve to liver cancer, which justifies the quest for a reliable biomarker that can be used at an early stage of the disease. The need for new guidelines based on biomarkers is highlighted by the controversial interest of alpha-fetoprotein (AFP) as a biomarker^25^^,^^26^ in association with ultrasound imaging of the liver at 6-month intervals. Exploring copper isotopic variations in liver tissues and blood is therefore relevant to the understanding of hepatic function because the liver is the largest reservoir of copper in the body and has a turnover of about 20 days,^27^ commensurate with the evolution rate of multiple pathologies, in particular cancer.

Pilot studies of copper isotope composition as a biomarker for liver^13^^,^^14^^,^^19^ involved about a few tens of patients and controls. Such a sample size is too small to warrant the robustness of statistical calculations.^28^, ^29^, ^30^ The present study includes larger numbers of patients with NAFLD etiologies,^31^ the milder NAFLD, with no evidence of hepatocellular injury, and the nonalcoholic steatohepatitis (NASH) with the presence of inflammation and hepatocyte injury. It also includes patients with HCC and a control group of 45 adults and 60 teenage individuals. A few patients with less common etiologies, such as primary biliary cirrhosis and primary sclerosing cholangitis, were included, but the data are given for reference only.

## Material and Methods

### Samples

The present study focuses on a survey of 293 patients from a French cohort dedicated to HCC investigation and stored at the INSERM CRCL Hépatologie biobank. Controls comprise a group of 45 adult blood donors provided by Lyon Etablissement Français du Sang (EFS) (22 men and 23 women, Table 1). Sixty samples were drawn from healthy teenage controls (27 men and 33 women) from the French VITADOS study (NCT01832623) targeted at a cross-sectional evaluation of bone, vessels, nutrition, and vitamin D status in healthy children and teenagers aged 10–18 years.^32^ All samples from these 3 cohorts are from individuals living within a close perimeter around Lyon, France. Samples from patients with NAFLD were obtained from Clinique Saint-Michel in Toulon (18–71 year old) with body mass index values of 31–52, that is, in the obesity range.

**Table 1:** Dataset Statistics

Parameter	Teenage controls				Adult controls			
	N	Minimum	Maximum	Median	N	Minimum	Maximum	Median
Cu (μg/L)	60	576	1408	905	45	733.1	2307.19	1067.6
δ^65^Cu	60	−0.63	0.28	−0.11	45	−0.61	0.07	−0.11
Gender	Male = 27 (45%) Female 33 (55%)				Male = 22 (51%) Female 22 (49%)			

Parameter	NAFLD patients				Other etiologies			
Age	52	18	71	42	293	17	86	57
Cu (μg/L)	55	865.52	2841.77	1664.95	291	334.48	1784.46	803.76
δ^65^Cu	55	−0.79	0.19	−0.3	293	−1.73	0.3	−0.41
AFP					245	0	25,400	6
Age at first HCC diagnosis					95	20	89	61
ALAT	39	9	246	25	280	14	247	45.5
ALB					194	4	830	41
ASAT	38	10	121	22	275	8	244	34
Bilirubin	12	3.6	19	5.95	281	2	343	11
Child-Pugh score					99	5	60	5
Creatinin uMA	46	4.9	41.8	6.95	269	39	531	71
GGT	35	6	151	23	276	2	1238	84.5
MELD Na					236	0.02	40	7.22
Overweight	41	31.2	51.8	38.9	118	16.5	40	25.78
PAL					273	16	943	86
Platelets (giga/L)	55	69	544	244	241	1	685	188
Prothrombin (%)	53	80	100	99	263	1	252	100
Gender	Male = 14 (25%) Female = 41 (75%)				Male = 190 (65%) Female = 102 (35%)			

AFP, alpha-fetoprotein; ALAT, alanine-amino-transférase; ALB, albumin; ASAT, aspartate-amino-transférase; GGT, gamma GT; HCC, hepatocellular carcinoma; MELD, model for end-stage liver disease; NAFLD, nonalcoholic fatty liver disease; PAL, poly-L-lysine induced agglutination of lymphocytes.

In both HCC-positive and HCC-negative groups, other etiologies are represented, notably hepatitis B virus and hepatitis C virus chronic infection, alcohol uptake (OH), and finally, cirrhosis. Liver pathology serum samples were taken during consultations for liver pathologies. The number of patients in the HCC-positive group includes 98 patients, 14 women and 84 men, with ages ranging from 31 to 85 years and a mean age of 56 years. The HCC-negative group (195 in total) consists of 89 women and 106 men, with ages ranging from 17 to 86 years and a mean age of 53 years. The breakdown of the patients according to chronic liver etiologies is summarized in Table 2. A number of biological constants monitoring liver functions such as fibrosis score, AFP (normal < 8 ng/mL), gamma GT (normal < 85 IU/L), aminotransferase (normal < 37 IU/L), alanine aminotransferase (normal < 78 IU/L), cirrhosis status, alkaline phosphatase (normal < 136 IU/L), and serum albumin (normal > 35 g/L) were also recorded.

**Table 2:** Breakdown of the Dataset by Etiology

Etiology	Number and percentage of patients
Autoimune	4 (1.4%)
HBV	41 (14%)
HCV	130 (44%)
Hemochromatosis	9 (3.1%)
NASH	25 (8.5%)
Alcoholic (OH)	57 (19.5%)
OH + HCV	13 (4.4%)
PBC	7 (2.4%)
PSC	1 (0.34%)
HBV + HCV	1 (0.34%)
HBV + NASH	1 (0.34%)
HCV + autoimune	2 (0.7%)
NASH + OH	1 (0.34%)
NASH + HBV	1 (0.34%)
Controls	45 (adult) + 60 (teenage)
NASH Clinique St Michel	55

HBV, hepatitis B virus; HCV, hepatitis C virus; NASH, nonalcoholic steatohepatitis; PBC, primary biliary cirrhosis; PSC, primary sclerosing cholangitis.

Fifty-five NASH serum samples from obese but HCC-negative patients (body mass index > 30) obtained before bariatric surgery were provided by the Clinique St Michel in Toulon, France, but the available parameters recorded were different from those used to monitor the previous group.

### Analytical Techniques

The serum samples were prepared at the INSERM U1052 laboratories classified at biosafety level 3 (P3) for sampling and acid addition before chemical separation of Cu and Cu isotopic analysis were carried out at ENS-Lyon. They were processed according to a protocol described elsewhere^33^ adapted to biological samples.^10^ All Cu separations were done in a clean laboratory environment. The samples were treated with 1 mL of concentrated bidistilled HNO_3_ + 0.2 mL of H_2_O_2_ (30%) in Savillex tubes at 125 °C. A 0.1-mL aliquot was taken for elemental analysis after digestion and before evaporation to dryness. Two milliliters of AG-MP-1 resin was used for Cu separation which was repeated to improve removal of interfering ions. The total procedural Cu blank represents less than 0.05% of the sample size.

Copper concentrations were measured on a single-quadrupole iCAP-Q or a triple-quadrupole Triple Quadrupole-Inductively Coupled Plasma-Mass Spectrometer (both Thermofisher Scientific), while Cu isotopic compositions were measured on a Nu HR Plasma I (Nu-Instruments, Wrexham) operated in low-resolution mode. The operational settings of each instrument can be found in the study by Telouk et al.^15^ The average standard reproducibility was better than 0.05‰.

### Statistical Methods

A first set of calculations and graphics was generated using MedCalc statistical software, version 18.6 (MedCalc Software bvba, Ostend, Belgium). For normality tests, quantitative parameters were compared between the different groups using a Student *t*-test. For non-normal populations, the nonparametric Mann-Whitney rank-sum test was used. The Wilcoxon test was used for the paired resection samples.

Coexisting populations of δ^65^Cu values were identified in 2 ways. First, the empirical probability density functions (*pdf*, or histograms) and cumulative density functions (*cdf*) were established. The *cdf* were fitted with Gaussian mixtures using the Matlab *fitgmdist* function which implements the iterative maximum-likelihood *Expectation-Maximization* (EM) algorithm. Mixtures of 2, 3, and 4 gaussian components were tested using the Pearson's chi-squared test. The *pdf*’s are derived from the parameters of the fitted *cdf*. Second, samples were assigned to the same number of clusters using the *clusterdata* function of Matlab which uses the Ward hierarchical linkage method. The detailed description of these functions and references to the underlying algorithms can be found on the Matlab website.

## Results

The mean value of δ^65^Cu in the sex-pooled control group (−0.21‰) is consistent with the data published previously.^10^ The average δ^65^Cu of adult (−0.17 ± 0.17‰, 95%) and teenage (−0.13 ± 0.20‰, 95%) controls cannot be separated. There is no significant gender difference for children (*P* = .39), whereas serum δ^65^Cu is higher for adult men relative to women (*P* = .05).

Overall, δ^65^Cu values are correlated with other relevant parameters (Table 3). AFP, which has been widely used as an HCC biomarker,^34^ is statistically significant and, on average, higher for HCC-positive patients (*P* < .0001). No significant correlation was observed between the MELD-Na score calculated with the Mayo Clinic formulae and δ^65^Cu (*P* = .62). There is no significant separation between patients who already entered cancer therapy and new HCC-positive patients.

**Table 3:** Correlation Between Biological Constants and δ^65^Cu and *t*-Test on Values of the Biological Constants Between HCC-Positive and HCC-Negative Patients

Biological constant (missing data)	*P* value (correl with δ^65^Cu)	*P* biological constant (HCC-positive vs negative)
AFP > 8 (45)	.0016	<.0001
AST > 37 (6)	.0055	<.0001
ALT > 78 (5)	.0053	.027
ALP > 136 (5)	.025	<.0001
GGT > 85 (3)	<.0001	<.0001

AFP, alpha-fetoprotein; ALP, alkaline phosphatase; ALT, alanine aminotransferase; AST, aminotransferase; GGT, gamma GT.

**Figure 1:**
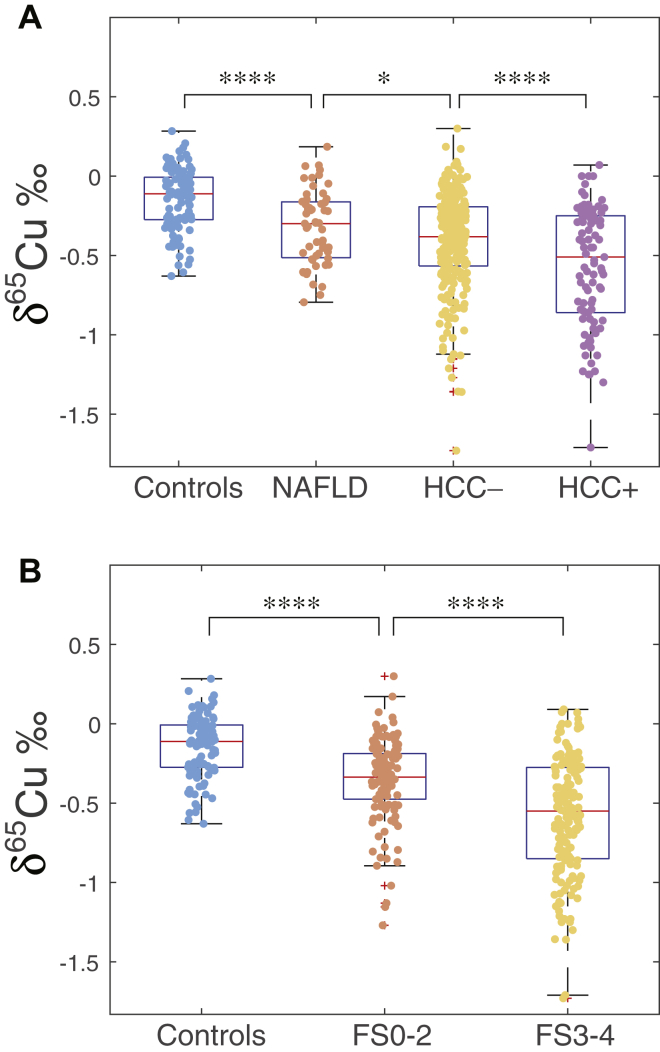
Comparison of δ^65^Cu serum values for (A) controls, NAFLD (inflammation), HCC-negative liver injuries, and HCC-positive patients; (B) controls, fibrosis scores F0-F1-F2, and F3-F4. HCC, hepatocellular carcinoma; NAFLD, nonalcoholic fatty liver disease.

Figure 1 shows a clear decrease of δ^65^Cu in serum with the progressive breakdown of liver functions:

i. δ^65^Cu values decrease significantly (*P* < .001) from controls to patients with NAFLD to HCC-negative and HCC-positive groups (Figure 1A).
ii. Two groups of fibrosis scores were considered, F0-F1-F2 and F3-F4. The correlation of δ^65^Cu with the fibrosis score (Figure 1B) is highly significant (*P* < .001 from controls to the F0-F1-F2, and *P* < .0001 from F0-F1-F2 to F3-F4).^18^
iii. δ^65^Cu is significantly lower for the NAFLD (bariatric) group than for the control group (*P* = .0004).^20^

Seric δ^65^Cu values of obese patients fall within the same range as other HCC-negative patients but are higher than those of HCC-positive patients and are in good agreement with those obtained by Hastuti et al.^35^

**Figure 2:**
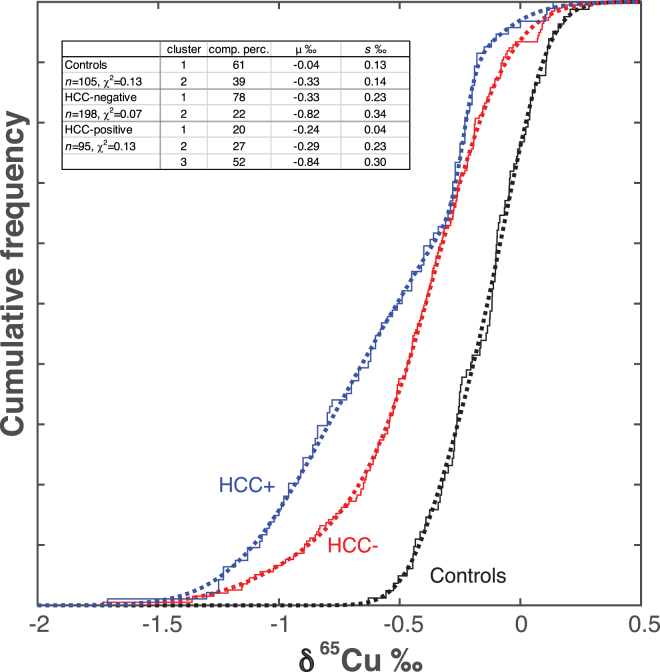
Cumulative distribution functions (*cdf*) of serum δ^65^Cu values for controls and HCC-negative and HCC-positive patients. The figure shows the actual (stair plots) and the fitted (dotted lines) *cdf*’s. A mixture of 3 normal populations (clusters) shows the best fit for the HCC-positive group. Although the data on HCC-negative patients are best interpreted by a mixture of 2 clusters, the break in the δ^65^Cu data *cdf* of HCC-positive patients at δ^65^Cu ∼−0.3‰ is striking. The peak of maximum frequencies at about −0.3‰ (inset) is found in each population. The frequency peak at ∼−0.84‰ increases with the transition from 0% for the controls to 22% for the HCC-negative patients and to 52% for the HCC-positive patients. The presence of this peak in the group of HCC-negative patients shows that it does not appear as a side effect of chemotherapy, nor of any other therapy directed against HCC. HCC, hepatocellular carcinoma.

**Figure 3:**
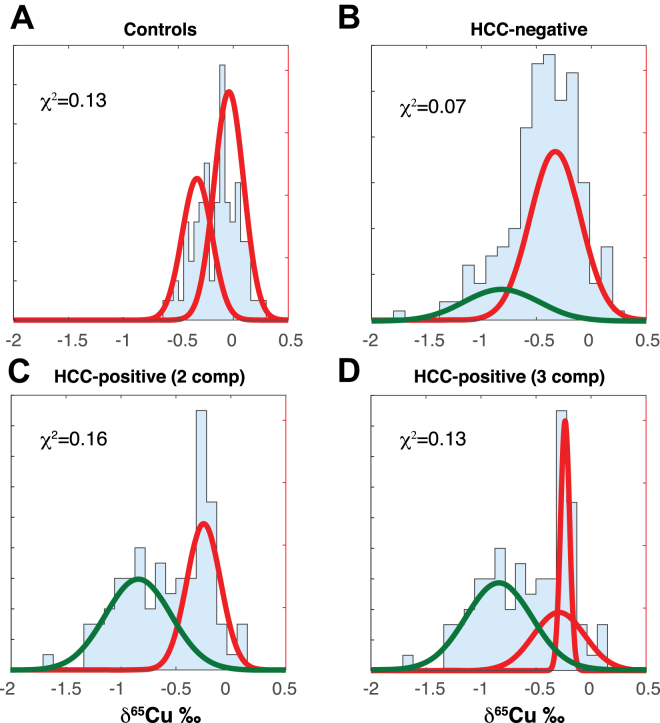
Comparison of the *pdf* derived from the fitted δ^65^Cu *cdf* assuming that each group considered is a mixture of 2 or 3 normal populations (components) with the observed δ^65^Cu histograms: teenage + adult controls (A), HCC-negative (B), and HCC-positive patients (C). A fit for HCC-positive patients (D) as a mixture of 3 components provides a better description of the data, in particular around δ^65^Cu ∼−0.3‰. Different colors refer to normal (red) and abnormal (green) δ^65^Cu values. The share of the cluster at ∼−0.84‰ (in green) increases from 0% for controls to 22% for HCC-negative patients and to 52% for HCC-positive patients. The samples are assigned to the different populations by cluster analysis, a technique consistent with the normal mixture of Figure 2. The χ^2^ values measure the quality of the fit by binary (controls, HCC-negative, and HCC-positive) and ternary (HCC-positive) mixtures of normal populations. HCC, hepatocellular carcinoma.

Figure 2 shows the results of the fit of the cumulated frequencies of the control, HCC-negative, and HCC-positive populations by a mixture of 2 and 3 normal *cdf’*s. Note the growth of a population with an average δ^65^Cu of −0.84‰. Figure 3 shows the corresponding *pdf*’s.

## Discussion

The present results confirm the preliminary observation^12^^,^^13^^,^^18^^,^^24^ that average serum δ^65^Cu progressively decreases upon breakdown of hepatic functions. This feature is enhanced for HCC-positive patients. Low sensitivity motivated the removal of AFP from HCC screening and diagnosis, but a combination of AFP levels with ultrasound has been found to improve sensitivity of early HCC detection.^36^ The present results show that δ^65^Cu is low when one or more of these constants are outside the normal range. They also echo the lower δ^65^Cu values observed for patients with Wilson disease with F3-F4 fibrosis scores with respect to patients with F0-F2 scores.^18^

The present study shows that the robustness of Cu isotopes as cancer biomarker is impaired by the coexistence in each sample group of more than one δ^65^Cu population. This is best illustrated by comparing the observed and fitted δ^65^Cu *cdf* for the different groups (Figure 2). Controls and HCC-negative and HCC-positive samples were merged irrespective of sex (M + F), and the data pooled within each group. Given the results on single-sex groups of data, each of the pooled groups was fitted by a mixture of 2 normal populations. As indicated by the χ^2^ values, the fit provided for the δ^65^Cu values by the code is excellent.

As visible by plotting the fitted *pdf* derived from the *cdf* parameters, the fit for controls is optimum for a mixing of 2 normal clusters (χ^2^ = 0.13), but the separation between the 2 groups (−0.04 and −0.33‰, *P* = .28) is marginal (Figure 3). A slight gender-related shift is also observed for the controls, but again the difference is not significant.

In contrast, for the HCC-negative group (χ^2^ = 0.07), the 2 peaks (−0.82‰ and −0.33‰, *P* = .0018) are significantly separated. The fit of the HCC-positive group is satisfactory with 2 normal clusters (χ^2^ = 0.16) but is visibly improved (χ^2^ = 0.13) when a third cluster is allowed for. The improvement is mostly noticeable at high δ^65^Cu values. A sharp frequency break is observed at δ^65^Cu ∼−0.3‰ (Figure 2). Cluster #3 peaking at δ^65^Cu ∼−0.84‰ is well separated (*P* = .08) from clusters #1 and #2 (−0.24‰ and −0.29‰, respectively), which separate themselves from each other by different standard deviations.

The δ^65^Cu shift between the HCC-negative and HCC-positive populations is similar. Frequency maxima at δ^65^Cu ∼−0.3‰ are consistent with the average value of M + F controls. For HCC-negative patients, a 22% frequency peak (#2) is observed at δ^65^Cu ∼−0.82‰ (green color in Figure 3), which is absent for controls. The peak increases to 52% for HCC-positive patients (#3), but its position at δ^65^Cu ∼−0.84‰ hardly changes. Nevertheless, the δ^65^Cu values of 78 HCC-negative patients and 48 HCC-positive patients remain within the range of the controls. This suggests that for these 126 patients, both HCC-negative (#1) and HCC-positive (#1 and #2), the hepatic function was essentially unimpaired in spite of inflammation, cirrhosis, and HCC.

The results obtained on NAFLD samples, which serve as a reference for inflammation-free patients, show a decrease in δ^65^Cu with respect to the control groups but not as pronounced as for patients with HCC (*P* < .001). As previously suggested for the δ^65^Cu data on patients with rheumatoid arthritis,^37^ inflammation is unlikely to represent the leading factor for triggering the δ^65^Cu variations observed in the serum of patients with cancer. The present results indicate that, in general, anomalous liver tissue produced by NAFLD, NASH, OH, and hepatitis B and C accounts for a substantial fraction of the Cu being present in the organism and therefore affects the overall Cu balance of the serum. This effect is significantly stronger for neoplastic tissues.^12^

A second remarkable outcome of the present study is that the serum of some HCC-negative and HCC-positive patients keeps δ^65^Cu values indistinguishable from those of the controls. In other words, the Cu status of several severely ill patients indicates that some apparently normal hepatic functions may be preserved in spite of HCC. This puzzling observation suggests that, for some patients with HCC, parts of the liver mass keep functioning and succeed in maintaining an apparently normal Cu status.

We interpret the present results as due to local variabilities in the oxidative stress on the organ well before HCC sets in. Copper (and Zn) homeostasis is known to be disrupted by various types of cancer (eg, Télouk et al.^15^), which is the motivation behind research on copper chelation therapy.^38^ A simple mass-balance (Cu conservation) assessment using concentration and δ^65^Cu data on serum and neoplastic tissues is unfortunately limited by the intrinsically open nature of homeostasis and the lack of clinical data on absorption and excretion fluxes. In vitro experiments actually demonstrated that hypoxia causes preferential enrichment of ^65^Cu in several human cell lines.^24^ Hypoxia-inducible factors (HIFs) regulate the transcription of genes involved in cellular metabolism, inflammation, angiogenesis, and proliferation.^39^ Nevertheless, the hypoxic ^65^Cu enrichment by HepG2 cells is pH, HIF-1, and HIF-2 independent.^24^ Because the liver acts as a filter for blood, this organ is usually the first to be invaded and become metastatic.^40^ The present study therefore suggests that the liver of some patients maintains, at least for an extended period of time, enough oxygen in the liver tissue and keeps Cu status apparently normal, whereas the liver of other patients loses its potential to control blood oxygenation at an early stage.

The striking isotopic dichotomy revealed in this work differs from a simple broadening of a single distribution of δ^65^Cu values and should draw attention to a possible switch of etiology and a breakout of HCC. Although the present study shows that anomalous δ^65^Cu values are not specific to HCC, it suggests that the resistance of the liver to multiple diseases, in particular to HCC, revolves around the overall stability of oxygenation in the organ tissue.

As a final remark, applying the population mixture approach to other parameters, such as AFP, was unsuccessful.

## Conclusions

The potential of Cu isotopes as a biomarker of hepatic function status was tested against a number of etiologies, such as obesity, fibrosis, cirrhosis, and HCC. Although, for a given patient, copper isotopes are not a robust biomarker of HCC, evidence for dual populations of serum δ^65^Cu for both HCC-negative and HCC-positive patients reveals that the oxidative stress experienced by hepatic tissues paves the way to a breakdown of normal hepatic functions and further to HCC oncogenesis.
